# Remodeling of the gut microbiome by *Lactobacillus johnsonii* alleviates the development of acute myocardial infarction

**DOI:** 10.3389/fmicb.2023.1140498

**Published:** 2023-03-08

**Authors:** Xinqin Zhong, Yucui Zhao, Lu Huang, Jiarui Liu, Kaiyue Wang, Xiumei Gao, Xin Zhao, Xiaoying Wang

**Affiliations:** ^1^Ministry of Education Key Laboratory of Pharmacology of Traditional Chinese Medical Formulae, Tianjin University of Traditional Chinese Medicine, Tianjin, China; ^2^State Key Laboratory of Component-based Chinese Medicine, Tianjin University of Traditional Chinese Medicine, Tianjin, China; ^3^School of Chinese Materia Medica, Tianjin University of Traditional Chinese Medicine, Tianjin, China

**Keywords:** *Lactobacillus johnsonii*, acute myocardial infarction, gut microbiome remodeling, serum metabolic biomarker, alleviation the development

## Abstract

**Introduction:**

The gut microbial community, which can be disturbed or repaired by changes in the internal environment, contributes to the development of acute myocardial infarction (AMI). Gut probiotics play a role in microbiome remodeling and nutritional intervention post-AMI. A newly isolated *Lactobacillus johnsonii* strain EU03 has shown potential as a probiotic. Here, we investigated the cardioprotective function and mechanism of *L. johnsonii* through gut microbiome remodeling in AMI rats.

**Methods:**

A rat model of left anterior descending coronary artery ligation (LAD)-mediated AMI was assessed with echocardiography, histology, and serum cardiac biomarkers to evaluate the beneficial effects of *L. johnsonii*. The immunofluorescence analysis was utilized to visualize the intestinal barrier changes. Antibiotic administration model was used for assessing the gut commensals’ function in the improvement of cardiac function post-AMI. The underlying beneficial mechanism through *L. johnsonii* enrichment was further investigated by metagenomics and metabolomics analysis.

**Results:**

A 28-day treatment with *L. johnsonii* protected cardiac function, delayed cardiac pathology, suppressed myocardial injury cytokines, and improved gut barrier integrity. The microbiome composition was reprogrammed by enhancing the abundance of *L. johnsonii*. Microbiome dysbiosis by antibiotics abrogated the improvement of cardiac function post-AMI by *L. johnsonii*. *L. johnsonii* enrichment caused remodeling of gut microbiome by increasing the abundance of *Muribaculaceae*, *Lactobacillus*, and decreasing *Romboutsia*, *Clostridia* UCG-014, which were correlated with cardiac traits and serum metabolic biomarkers 16,16-dimethyl-PGA2, and Lithocholate 3-O-glucuronide.

**Conclusion:**

These findings reveal that gut microbiome remodeling by *L. johnsonii* ameliorates the cardiac function post-AMI and might advance microbiome-targeted nutritional intervention.

**Graphical Abstract:**
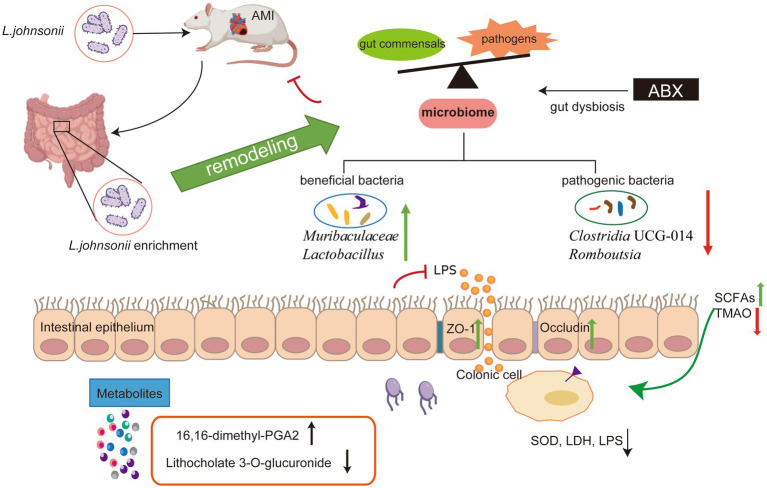

## 1. Introduction

Cardiovascular diseases (CVDs) are serious health and social problems (Fuster, 2014). More than 50% of deaths related to CVDs are due to acute myocardial infarction (AMI), and therapeutic strategies for reducing the risk of AMI, including pharmacological and surgical interventions, still lack clear prophylactic measures that integrate the evolutionary and cardiac biomarkers of AMI (Pollard, 2000). Growing evidence shows that the gut microbiota is associated with cardiac repair processes and may be involved in ventricular remodeling after AMI (Rogler and Rosano, 2014; Tang et al., 2017; McMillan and Hazen, 2019). The gut microflora of patients with AMI contains a lower abundance of the phylum Firmicutes and slightly higher abundance of the phylum Bacteroidetes than those of healthy controls (Han et al., 2021). The disruption of gut micro-community triggers AMI and results in poor prognosis, and the gut microbiota is readily altered by various interventions targeting host signaling pathways involved in AMI pathogenesis (Zununi Vahed et al., 2018; McMillan and Hazen, 2019). Remodeling is an alternative approach for exploring the nutritional potential of the gut microbiome. Given the plasticity of microbiota to changes in the chemical milieu within the gut, a wide range of probiotics, natural products, or prescribed therapeutics, potentially remodel the gut microbiota (Chen et al., 2020).

As gut commensals, the effectiveness of *Lactobacillus* sp. strains in preventing and treating CVDs in clinics has been extensively studied (Zhao et al., 2021b), such as *L. acidophilus* La5, *L. amylovorus* CP1563, *L. casei* Shirota, *L. fermentum* ME-3, *L. gasseri* BNR17, *L. helveticus* LBK-16H, *L. plantarum* Lp299v, *L. reuteri* NCIMB30242, *L. rhamnosus* GG, *L. sakei* CJLS03, and *L. salivarius* Ls-33, etc. Notably, *L. rhamnosus* has been effectively used in attenuating cardiac remodeling after AMI (Moludi et al., 2019). The colonization and enrichment of *Lactobacillus* in the gut may be a new approach for the improvement of cardiac function post-AMI. Dietary supplements, especially natural products, promote the growth of *Lactobacillus* (Zhao et al., 2021a). Specific strains and metabolites as interference targets for gut microbiome remodeling should be further explored.

A *Lactobacillus johnsonii* strain EU03 was isolated from a fecal sample with antimicrobial activity. To investigate the effect of *L. johnsonii* enrichment on gut microbiome and cardiac function after AMI, we detected echocardiographic evaluation, cardiac biomarkers, gut microbiota composition, and serum metabolites in rats. The stimulating effects of *Lactobacillus* on the gut microbiota may help explain its amelioration efficacy. Here, we elucidate the beneficial effect of gut microbiome remodeling by *L. johnsonii* on alleviating the development of AMI *in vivo*.

## 2. Materials and methods

### 2.1. Microbiological manipulation

Strains used in this study were preserved at the Microbiology Lab of Tianjin University of Traditional Chinese Medicine. *L. johnsonii* strain EU03 was isolated from fresh C57BL/6J mouse fecal sample and identified as *L. johnsonii* with probiotic potential. The genomic DNA of strain EU03 was further sequenced using Pacific Biosciences RS sequencing technology (Pacific Biosciences, Menlo Park, CA) by Novogene Co., Ltd. (Beijing, China) and has been deposited in Genbank, under the BioProject accession number PRJNA732116. *L. johnsonii* strain EU03 is deposited in the China Pharmaceutical Culture Collection (CPCC) under accession number CPCC101288 and China General Microbiological Culture Collection Center (CGMCC) under accession number CGMCC20845 (Supplementary Figure S1). The reference strain, *Lactobacillus rhamnosus* strain BNCC134266 (ATCC7469 = CCRC10940 = CGMCC1.2436 = DSM20021), was purchased from commercially from BeNa Culture Collection Co., Ltd. (Beijing, China). *Lactobacillus* strains were activated by transferring single colonies of the strain from plates to 10 mL activation MRS medium (Solarbio, Beijing, China) extract in the 50-mL static flask at 37°C, for 24 h. The cells of these two strains were collected by centrifuged at 4,000 *g* for 15 min and resuspended in normal saline and then prepared to be the microbial agents of 10^8^ c.f.u./mL for further animal feeding.

### 2.2. Animals

Two-month-old female C57BL/6J mice were purchased from Huafukang Co., Ltd. (Beijing, China) and six-week-old Sprague–Dawley (SD) male rats were purchased from Weitong Lihua Animal Experiment Center (Beijing, China), and housed in the Animal Centre of Tianjin University of Traditional Chinese Medicine at a controlled SPF condition (22 ± 2°C, 40–60% humidity, 12 h light–dark cycle). This study was carried out in strict accordance with the recommendations in the Guidance Suggestions for the Care and Use of Laboratory Animals issued by the Ministry of Science and Technology of China and approved by the Laboratory Animal Ethics Committee of Tianjin University of Traditional Chinese Medicine (TCM-LAEC2019015).

### 2.3. Acute myocardial infarction model and drug administration

After a week of adaptation, the occlusion of the left anterior descending coronary artery (LAD) surgery was employed to induce AMI in rats. Isoflurane was purchased from Abbott Laboratories (Shanghai, China). The LAD was permanently fixed at 2 to 3 mm from the left atrial appendage (Rasanen et al., 2021). All experiment animals after surgery were randomly divided into groups (20 rats per group): (A) Sham group (without LAD), (B) AMI group (with LAD), (C) *Lactobacillus*-treated groups (LJ or LR), and (D) antibiotics-treated groups (ABX).

The rats in the groups A and B received the same volume of saline. The rats in the groups C were treated with the bacterial culture of *L. johnsonii* or *L. rhamnosus* at a concentration of approximately 10^8^ c.f.u./mL. The bacteria and saline were orally administered once per day. The antimicrobial activity of antibiotics against *L. johnsonii* or *L. rhamnosus* was detected and presented in Supplementary Figure S2. For *in vivo* antibiotics treatment in the groups D, AMI rats were treated with either *Lactobacillus*, or saline, combined with antibiotics cocktail prepared as previously, including 62.5 μg/mL ampicillin, 62.5 μg/mL metronidazole, 62.5 μg/mL neomycin, and 31.25 μg/mL vancomycin in sterile water (Tang et al., 2019). ABX treatment was initiated 7 days before AMI surgery.

### 2.4. Echocardiographic evaluation and sample collection

To confirm the AMI status and evaluate the efficacies of bacteria or drugs, echocardiographic examinations were performed the second day after surgery and 7, 14, 28-day consecutive administration. All rats were anesthetized using isoflurane (complete anesthesia: 1% oxygen and 5% isoflurane in the anesthesia box; continuous anesthesia: 1% oxygen and 2% isoflurane) and a Vevo2100 Ultrasound equipped with an MS-250, 16.0–21.0 MHZ intraoperative probe (Visual Sonics, Canada) was employed for echocardiography. The left ventricular (LV) parameters were obtained from 2-dimensional images and M-mode interrogation in the long-axis view. The ejection fraction (EF) and fractional shortening (FS) were recorded, and the data were averaged over five consecutive cardiac cycles.

All rats were sacrificed under the deeply anesthetized condition after echocardiographic examinations. The collected serum samples were centrifuged at 1,000 *g* at 4.0°C for 15 min and stored at −80°C until analysis. The lower part of the myocardium for histopathological analysis was quickly collected and fixed in 10% formalin buffer for 48 h.

### 2.5. Pathological examination

#### 2.5.1. Myocardial damage detection

Myocardial tissue fixed in 10% formalin buffer was subjected to histopathological observation. The fixed myocardial tissue was embedded in paraffin and cut into 5 μm sections. Then the sections were stained with hematoxylin–eosin (H&E), wheat germ agglutinin (WGA), Sirius Red, and Masson as described previously (Emde et al., 2014; Kong et al., 2018; Varasteh et al., 2019; Liao et al., 2021).

#### 2.5.2. Colonic permeability detection

The colonic segments were cut out and fixed with phosphate-buffered formalin and embedded in paraffin. Then, the sections were stained with H&E and Alcian blue (AB). The changes of zonula occludens-1 (ZO-1) and occludin protein in colonic tissue were determined with immunofluorescence staining. Briefly, paraffin-embedded sections of colon tissue were prepared and incubated with antibodies according to Xie et al. (2021). Sections were counterstained with DAPI.

For all histological staining analyses, the pathological sections were photographed with a light microscope (Eclipse CI, Nikon Corporation, Tokyo, Japan) and an EVOS M7000 imaging system (Thermo Fisher Scientific, NJ, United States). The positive areas were quantified with the ImageJ software program (Bethesda, MD, United States).

### 2.6. Biochemistry assays

The contents of lactate dehydrogenase (LDH), creatine kinase isoenzymes (CK-MB), and cardiac troponin-T (cTnT), and the activity of malondialdehyde (MDA), the antioxidant superoxide dismutase (SOD), TNF-α, and LPS in serum were determined by corresponding ELISA kits. ELISA kits for cytokines detection were purchased from Lanpai Biological Technology Co., Ltd. (Shanghai, China) and Nanjing Jiancheng Institute of Biotechnology (Nanjing, China). All the procedures were performed according to the manufacturer’s instructions. These indicators of serum were determined by spectrophotometry (UV-3100, Mapada, China).

### 2.7. Fecal microbiota analysis

Fresh fecal samples (200 mg of each) were collected under sterile conditions and immediately frozen at −80°C. Genomic DNA was extracted using the E.Z.N.A.^®^ soil DNA Kit (Omega Bio-Tek, Norcross, GA, United States) according to the manufacturer’s protocols, and the concentration was detected by NanoDrop 2000 UV–vis spectrophotometer (Thermo Scientific, Wilmington, United States). The V3-V4 regions of the 16S rRNA gene were amplified with primers 338F (5′- ACTCCTACGGGAGGCAGCAG-3′) and 806R (5′-GGACTACHVGGGTWTCTAAT-3′; Liu et al., 2016), purified and quantified according to the manufacturer’s protocol. Purified amplicons were then paired-end sequenced on an Illumina MiSeq platform (Illumina, San Diego, United States). Raw reads were quality-filtered and merged by FLASH (Magoc and Salzberg, 2011). Operational taxonomic units (OTUs) were clustered by ≥97% similarity cutoff using UPARSE (version 7.1).^1^ The taxonomy of each 16S rRNA sequence was analyzed by the RDP Classifier algorithm^2^ and annotated by the Silva database. Chao1, ACE, Shannon, and Simpson index were generated for alpha diversity analysis and the bray curtis algorithm for Principal Coordinate Analysis (PCoA) was computed for beta diversity analysis. Correlations between the key microbial phylotypes and metabolites were calculated by Spearman correlation analysis.

### 2.8. SCFAs production analysis

Blood serum samples (each about 200 μL), after dilution with 100 μL of 15% phosphoric acid, 100 μL of 75 μg/mL of isohexanoic acid (internal standard), and 280 μL ether, were centrifuged at 4°C and 12,000 *g* for 10 min, and the supernatant was taken for gas chromatography-tandem mass spectrometry (GC–MS) analysis with Agilent hp-innowax capillary column 30 m × 0.25 mm × 0.25 μm; ion source temperature, 230°C; detector temperature, 250°C; carrier helium, 1.0 mL/min (Thermo Scientific, Wilmington, United States). MS condition was EI source, SIM scanning mode, electron energy 70 eV. Acetic acid, propionic acid, isobutyric acid, butyric acid, isovaleric acid, valeric acid, and caproic acid were detected as standard reference materials (Anpel Experimental Technology Co., Ltd., Shanghai, China).

### 2.9. TMAO related compounds quantitative analysis

Blood serum samples (each about 20 μL) were diluted with 750 μL of 1% formic acid acetonitrile solution with 10 μL of the internal standard including choline-d9 (5 μM), betaine-d9 (5 μM), TMAO-d9 (3 μM), creatinine-d9 (400 μM), L-carnitine-d9 (5 μM). After centrifuged at 4°C and 12,000 *g* for 5 min, 500 μL filtered supernatant was determined by ultra-performance liquid chromatography–tandem mass spectrometry (UPLC-MS) using an Acquity BEH Amide column (1.7 μm × 2.1 mm × 100 mm) at a flow rate of 0.4 ml/min (Waters, Milford, MA, United States). MS condition was ESI source, MRM scanning mode, under electron energy 5,000 V and ion source temperature 500°C. Choline, betaine, TMAO, creatinine, and L-carnitine were detected as standard reference materials (Zhenzhun Biotechnology, Co., Ltd., Shanghai, China).

### 2.10. Untargeted metabolomics analysis

The serum samples were thawed at 4°C, 400 μL extraction solution [methanol: acetonitrile = 1:1 (v: v)], containing 0.02 mg/mL internal standard (L-2-chlorophenylalanine) was added into the serum samples (100 μL). After placation at −20°C for 30 min and centrifugation for 15 min (13,000 *g*, 4°C), the supernatant was blowed dry with nitrogen and added 100 μL solution (acetonitrile: water =1:1). And the sample tubes were vortexed (30 s) and extracted with ultrasound (5 min, 5°C, 40 KHz). After centrifuged at 13,000 *g* for 10 min at 4°C, the resulting supernatant was analyzed by UPLC-TOF/MS carrying out on BEH C18 column (100 mm × 2.1 mm i.d., 1.8 μm; Waters, Milford, United States). The QC sample was prepared by mixing an equal volume (20 μL) aliquot from each serum sample. The MS was performed on a TripleTOF spectrometer equipped with an ESI source in both positive and negative ion scan modes, over a mass range of 50–1,000 m/z, at 500°C. The spray voltage was set at 5,000 V (+) and 4,000 V (−); the normalized collision energy was set at 20, 40, 60 eV. Leucine-enkephalin [m/z 556.2771 (+) and m/z 554.2615(−)] was used as the mass reference compound in the positive and negative ion mode, respectively.

MS/MS fragments spectra and isotope ratio difference with searching in reliable biochemical databases as human metabolome database (HMDB)^3^ and Metlin database.^4^ Further statistical analysis was performed using ropls (Version1.6.2)^5^ R package. Principle component analysis (PCA) and orthogonal partial least squares discriminate analysis (OPLS-DA) were conducted to determine global metabolic changes between comparable groups. Statistically significant among groups were selected with variable importance in the projection (VIP) value more than 1 and *p* value less than 0.05.

### 2.11. Statistical analysis

The experimental data are expressed as mean ± SEM ($\bar{x}$±s), and SPSS statistical software (version 20.0, SPSS Inc., Chicago, IL, United States) is used for data analysis. One-way analysis of variance (One-way ANOVA) was used to compare groups. When the variances were uniform, the LSD test was used, and when the variances were uneven, Dunnett’s T3 test was used. When *p* < 0.05, the test results are significantly different and have statistical significance.

## 3. Results

### 3.1. *Lactobacillus johnsonii* EU03 protects cardiac function and improves intestinal barrier after AMI

**Figure 1:**
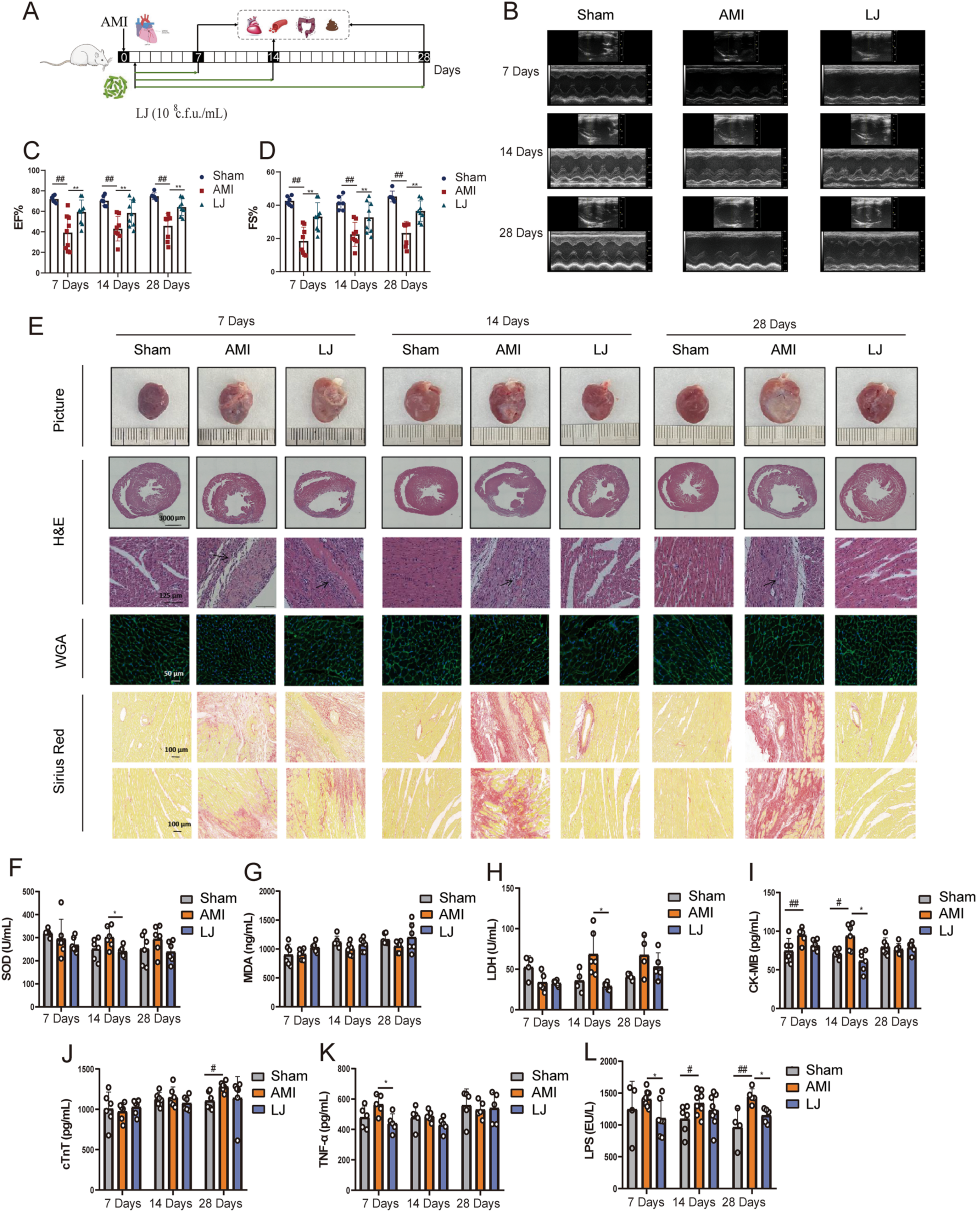
*Lactobacillus johnsonii* EU03 protects cardiac function after AMI. **(A)** The schematic diagram for *L. johnsonii* administration pipeline in rats after AMI. **(B)** Representative echocardiograph of rats left ventricle. **(C)** EF and **(D)** FS calculated using dimensional measurements of rats left ventricle. **(E)** Representative heart pictures, H&E staining [scale bar equals 3,000 μm (top) and 125 μm (bottom)], Wheat germ aggregates (WGA) staining (scale bar equals 50 μm), and Sirius Red staining (scale bar equals 100 μm). Serum levels of **(F)** SOD, **(G)** MDA, **(H)** LDH, **(I)** CK-MB, **(J)** cTnT, **(K)** TNF-α, and **(L)** LPS. ^#^*p* < 0.05, ^##^*p* < 0.01 versus Sham and ^*^p < 0.05, ^*^p < 0.01 versus AMI.

The microbial agent of *L. johnsonii* was administered to rats orally 7, 14, 28 days after AMI (Figure 1A). Echocardiography assessment indicated that LAD ligation markedly enlarged left ventricular volume and decreased EF and FS. *L. johnsonii*-feeding significantly enhanced myocardial echo, EF and FS levels (*p* < 0.01, vs. AMI) after 7, 14, 28 day-treatment, respectively (Figures 1B–D). The macroscopic images of the hearts revealed that the necrosis and hypertrophy in the model group were serious as the number of days increased. The prolonged administration of the *L. johnsonii* improved the macroscopic images of the hearts alike the sham-operated group (Figure 1E). Histopathological detection demonstrated that the longer the ligation time, the thinner of the LV wall accompanied by inflammatory cell infiltration, the more myocardial hypertrophy, and the more serious perivascular fibrosis and interstitial fibrosis were present in the AMI rats. The slight improvement of histopathological lesions was shown by 7-day administration of *L. johnsonii*, and obvious amelioration were observed after 14- and 28-day administration of *L. johnsonii*. The serum levels of cardiac markers SOD, LDH, CK-MB were elevated in the AMI group after 7, 14 days, and cTnT was elevated in the AMI group after 28 days. Oral administration of *L. johnsonii* for 7 days significantly decreased the level of TNF-α, *L. johnsonii* administration for 14 days significantly decreased the levels of SOD, LDH, and CK-MB (Figures 1F–K). The serum levels of SOD, LDH, and cTnT were decreased after28 days’ treatment, but with no significant difference. Serum LPS level was detected to evaluate the disruption of the intestinal barrier (Figure 1L). LPS levels increased gradually with time after AMI. Compared with model rats, *L. johnsonii* intervention had significantly lower LPS levels for 28 days.

**Figure 2:**
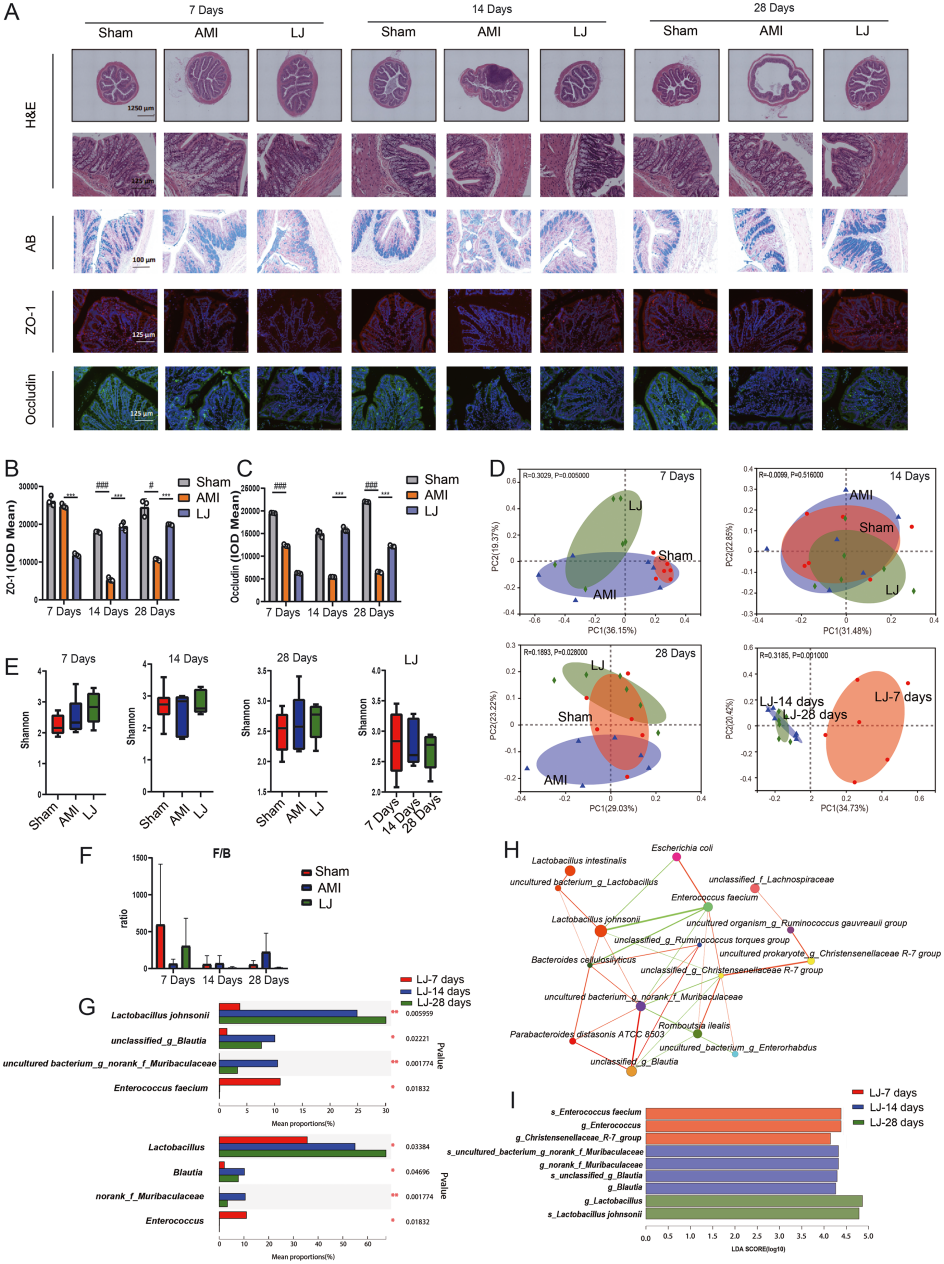
*L. johnsonii* EU03 improves intestinal barrier and affects the composition of gut microbiome after AMI. **(A)** Histopathological examination of colon tissue by H&E staining [scale bar equals 1,250 μm (top) and 125 μm (bottom)], Alcian blue (AB) staining (scale bar equals 100 μm), and representative immunofluorescence photomicrographs for tight junction proteins zonula occludentes (ZO)-1 (red) and occludin (green) in the colonic epithelium (scale bar equals 125 μm). **(B)** The ZO-1 and **(C)** occludin intensity were quantified by calculating integrated optical density (IOD). **(D)** Beta diversity of bray curtis-based PCoA of different groups for 7, 14, and 28 days administration, and different time plots together. Significant *p* values of Anosim between groups emphasize the differences in microbial community structure. **(E)** Alpha diversity of Shannon index in each group. **(F)** F/B ratio of different groups. **(G)** Kruskal-Wallis H test bar graph for specific microbial composition at genus and species levels. **(H)** Species correlation network at the species level. The correlation coefficient of Spearman between species was calculated to reflect the correlation between species. By default, the Figure shows the species with *p* < 0.05; the size of nodes indicates corresponding bacteria abundance, red line indicates positive correlation while the green line indicates negative correlation; a greater number of lines indicates closer relationship between species. **(I)** LEfSe at different levels of each group. The length of the histogram represents the abundance of the difference species (i.e., LDA score). “g for genus” and “s for_species.” ^#^*p* < 0.05, ^##^*p* < 0.01, ^###^*p* < 0.001versus Sham and ^*^p < 0.05, ^**^p < 0.01, ^***^p < 0.001 versus AMI.

In Figure 2A H&E-stained sections showed that the colonic mucosa of rats in the model group showed congestion, edema, ulceration, and a large amount of lymphocyte and neutrophil infiltration. However, the pathological damages of the colon caused by AMI were significantly improved in intervention with *L. johnsonii*. Compared to the sham group, AB staining of the colon in the model group displayed more seriously impaired in colonic goblet cells, which was partly improved by *L. johnsonii* treatment (Figure 2A). We also inspected the expression level of the major tight junction protein in the colon. As the biomarkers for intestinal barrier integrity, ZO-1 and occludin production in the model group gradually decreased with increasing days, and higher expression of them were found in the LJ group compared to that in the AMI group (Figures 2B,C).

### 3.2. *Lactobacillus johnsonii* EU03 remodels the gut microbiota after AMI

To determine the effect of *L. johnsonii* on the gut microbiota structure 7, 14, 28 days post-AMI, we conducted a bray curtis-based PCoA analysis. As shown in Figure 2D, the microbial community was not significantly changed in the 7- and 14-day model groups compared with the corresponding sham groups, while the microbial community was slightly altered after *L. johnsonii* administration; especially presenting stable after 14 days till 28 days. The richness of gut microbiota by analysis Shannon index fluctuated over the ischemic period and showed no significant difference between sham, AMI, and LJ groups (Figure 2E). At the phylum level, the ratio of Firmicutes to Bacteroidetes (F/B) in the gut of rats decreased after 7 days of AMI surgery and increased after the administration of *L. johnsonii*. In contrast, the ratio of F/B in the model group increased after 14- and 28-days treatment, and the F/B value in the LJ group gradually decreased and stabilized with the prolonged *L. johnsonii* administration (Figure 2F). Importantly, in the gut of the LJ groups, the enrichment of genus *Lactobacillus* increased with the extension of the administration time. The abundance of genus *Blautia*, *norank_f_Muribaculaceae* was increased, and the highest abundance was reached on the 14^th^ day. Besides, the abundance of genus *Enterococcus* was decreased on the 14th and 28th day (Figure 2G). Corresponding in species level, the abundance of *L. johnsonii*, *unclassified_g_Blautia, uncultured_bacterium_g_norank_f_Muribaculaceae*, and *Enterococcus faecium* were changed with the same trend. Moreover, the univariate network analysis of species reflected that *L. johnsonii* had the highest abundance in the gut microbial community, positively correlated with *Muribaculaceae* and negatively correlated with *Enterococcus* (Figure 2H). The enrichment and variations of bacterial community in each group were summarized by LEfSe in Figure 2I. *Enterococcus* was enriched in the 7-day treated group. *Blautia*, *norank_f_Muribaculaceae* were enriched in the 14-day treated group. *L. johnsonii* was enriched in the 28-day treated group (Figure 2I). These results showed *L. johnsonii* administration at 28 days post-AMI enriched the abundance *L. johnsonii* which paralleled with the alteration of gut microbiota and barrier.

### 3.3. Antibiotics induced dysbacteriosis interferes beneficial effect of *Lactobacillus johnsonii* post-AMI

**Figure 3:**
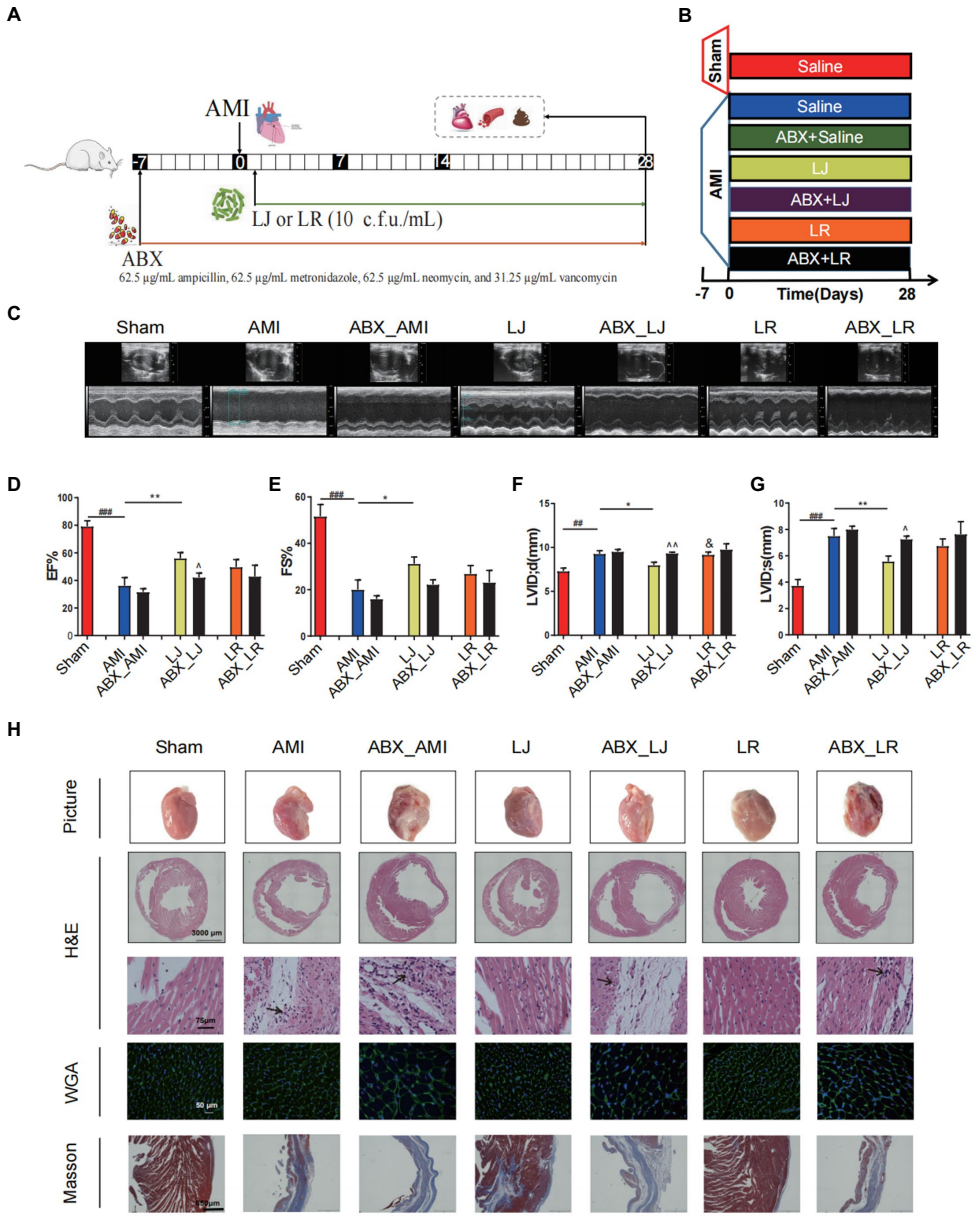
Antibiotics (ABX) induced dysbacteriosis interferes the beneficial effects on cardiac function of *L. johnsonii*. **(A)** The schematic diagram for *L. johnsonii* and ABX administration pipeline in AMI rats. **(B)** Rats were treated with ABX for 7 days prior to LAD surgery. The sham group and the AMI group were treated daily with control saline, *Lactobacillus* alone or simultaneously with ABX for 28 days by oral gavage. **(C)** Representative echocardiograph of rat’s left ventricle. **(D)** EF, **(E)** FS, **(F)** LVID; d and **(G)** LVID; s calculated using dimensional measurements of rats left ventricle. **(H)** Representative heart pictures, H&E staining [scale bar equals 3,000 μm (top) and 75 μm (bottom)], WGA and Masson staining of heart tissue (scale bar equals 50 μm and 650 μm). ^#^*p* < 0.05, ^##^*p* < 0.01, ^###^*p* < 0.001 versus Sham, ^*^p < 0.05, ^**^p < 0.01 versus AMI, ^&^*p* < 0.05 versus LJ and ^^^*p* < 0.05, ^^^^*p* < 0.01 versus AMI, LJ, LR, correspondingly.

To determine the importance of *L. johnsonii* and the alteration of gut microbiota in the amelioration post-AMI, we administered antibiotics to rats orally 7 days before AMI induction to inhibit *L. johnsonii* and disturb the microbiota (Figures 3A,B). *L. johnsonii* enrichment caused remodeling of gut microbiome by increasing the abundance of *Muribaculaceae*, *Lactobacillus*, and decreasing *Romboutsia*, *Clostridia* UCG-014 (Supplementary Figures S3A,B). ABX treatment had inhibitive effect on species of *Muribaculaceae* and *Lactobacillus*. Spearman’s correlation analysis indicated that the abundance of ABX sensitive bacteria *L. johnsonii* positively correlated with the levels of EF, FS, and SOD, while negatively correlated with MDA, LDH, and cTnT (Supplementary Figure S3C). Echocardiographic examination indicated that the *Lactobacillus* treatment improved the cardiac function, and ABX treatment did not affect the model samples (ABX-AMI) compared with the ABX-free model samples (AMI) after the 28 days of administration (Figure 3C). Notably, compared with the ABX-free administration LJ group, ABX treatment (ABX-LJ) abolished cardiac protection effects of *L. johnsonii*. The EF level was significantly lower in the ABX-LJ group compared with the LJ group (*p* < 0.05), and LVID; s and LVID; d were significantly higher (*p* < 0.05, *p* < 0.01). The comparisons between LJ and LR in the level of EF, FS and LVID; s were no differences, while the LVID; d level was significantly lower in the LJ group compared with the LR group (*p* < 0.05; Figures 3D–G). Observation of heart macroscopic pictures indicated that the necrotic area of the AMI group was larger compared with the sham group (Figure 3H). The necrotic area of the *L. johnsonii*-administered group was smaller than that of the model and the antibiotic groups, and the appearance of the hearts was as intact and smooth as that of sham group. Further histopathological analysis revealed that the ABX-LJ treatment did not improve the degree of interstitial edema and inflammatory infiltration of cardiomyocytes, with a thinner left ventricular wall, an increased lesion area, a certain degree of myocardial injury, and a large amount of interstitial fibroplasia. Similarly, the beneficial effects on cardiac function of *L. rhamnosus* administration were also reduced by ABX treatment.

### 3.4. Identification of the serum metabolic biomarker by *Lactobacillus johnsonii* enrichment

**Figure 4:**
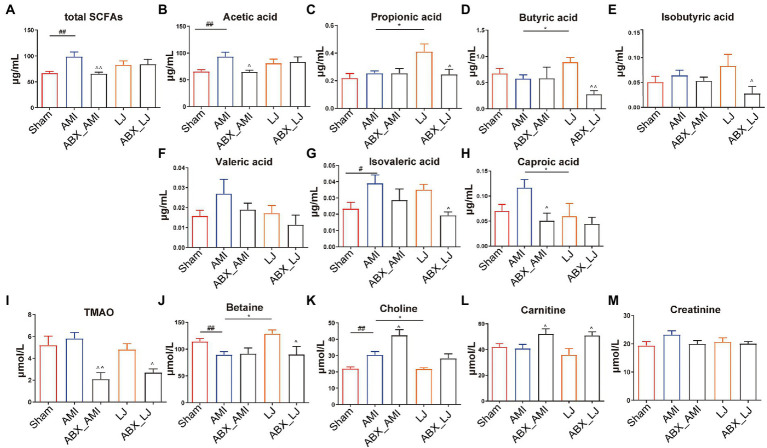
Serum levels of **(A–H)** SCFAs and **(I–M)** TMAO related metabolites in rats. ^#^*p* < 0.05, ^##^*p* < 0.01 versus Sham, ^*^p < 0.05 versus AMI and ^^^*p* < 0.05, ^^^^*p* < 0.01 versus AMI, LJ, correspondingly.

We tested the serum metabolite profiles of *L. johnsonii* treatment associated with the gut microbiota. The serum levels of total SCFAs, acetic acid, isovaleric acid and choline were elevated in the AMI group, compared with the Sham group (*p* < 0.05, *p* < 0.01), while the level of betaine was decreased in the AMI group (*p* < 0.01). Compared with the model group, LJ could significantly increase the content of serum propionic acid, butyric acid, betaine, and decrease the content of caproic acid, choline (*p* < 0.05). And compared with the LJ group, ABX treatment could significantly inhibit the level of SCFAs and TMAO related metabolites, including propionic acid, butyric acid, isobutyric acid, isovaleric acid and betaine (*p* < 0.05, *p* < 0.01) (Figures 4A–M).

**Figure 5:**
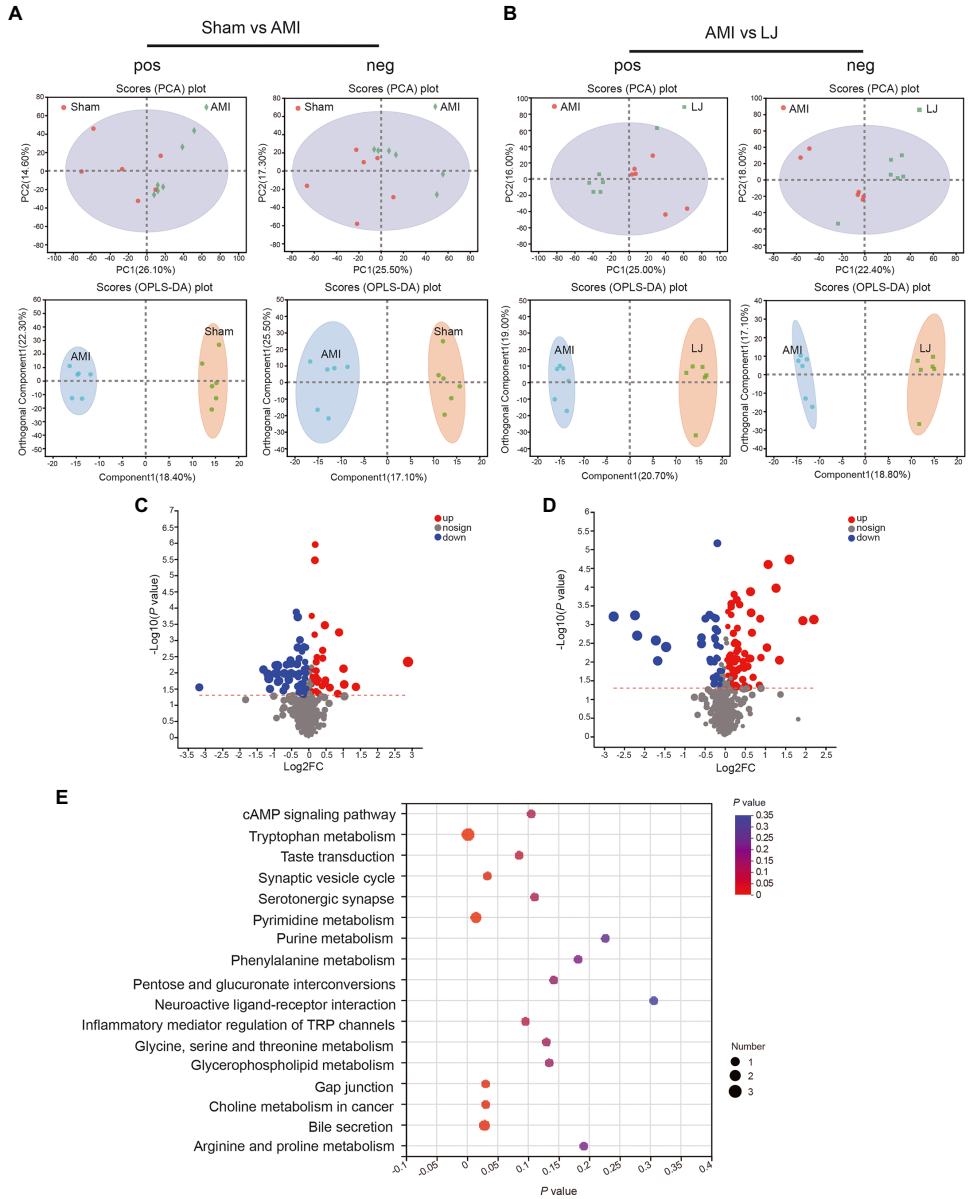
Circulating metabolomics for the quantification of metabolites among different groups. **(A)** The PCA score plots between Sham and AMI in positive ion mode [R^2^Xp1 = 0.261, R^2^Xp2 = 0.146, R^2^Xp3 = 0.13] and negative ion mode [R^2^Xp1 = 0.255, R^2^Xp2 = 0.173, R^2^Xp3 = 0.136]. The OPLS-DA score plots between Sham and AMI in positive ion mode [R^2^X = 0.184, R^2^Y = 0.7, Q^2^ = 0.475] and negative ion mode [R^2^X = 0.171, R^2^Y = 0.717, Q^2^ = 0.45]. **(B)** The PCA score plots between AMI and LJ in positive ion mode [R^2^Xp1 = 0.25, R^2^Xp2 = 0.16, R^2^Xp3 = 0.115] and negative ion mode [R^2^Xp1 = 0.224, R^2^Xp2 = 0.18, R^2^Xp3 = 0.135]. The OPLS-DA score plots between AMI and LJ in positive ion mode [R^2^X = 0.207, R^2^Y = 0.837, Q^2^ = 0.607] and negative ion mode [R^2^X = 0.188, R^2^Y = 0.876, Q^2^ = 0.582]. Volcano plot between **(C)** Sham and AMI, **(D)** AMI and LJ showing the differentially accumulated [log2 (fold-change) on *x*-axis] and significantly changed [−log10 (*p* value) on *y*-axis] metabolites. **(E)** Pathway enrichment based on altered metabolites after LJ administration.

To further investigate the global metabolism variations of the bio-candidates by *L. johnsonii* associated with the gut microbiota, we conducted an untargeted metabonomic analysis. A remarkable separation between the Sham and AMI samples was also observed in the supervised OPLS-DA score plot, as well as a significant separation of samples between Sham and AMI groups in the PCA score chart. *L. johnsonii* group also showed a remarkable separation compared with the AMI group (Figures 5A,B). Compared with the Sham treated group, AMI group showed 59 differential metabolites were detected in pos mode, and 37 differential metabolites in neg mode. Correspondingly, LJ group showed 81 differential metabolites were detected in pos mode, and 36 differential metabolites in neg mode after AMI (Figures 5C,D). Amongst the differential metabolites caused by AMI, a total of 60 differential metabolites were corrected after LJ administration (Supplementary Table S1). KEGG enrichment analysis reflected that LJ-affected compounds were involved in the cAMP signaling pathway, tryptophan metabolism, taste transduction, synaptic vesicle cycle, pyrimidine metabolism, purine metabolism, phenylalanine metabolism, neuroactive ligand-receptor interaction, inflammatory mediator regulation of TRP channels, glycine, serine and threonine metabolism, glycerophospholipid metabolism, and choline metabolism in cancer (Figure 5E).

**Figure 6:**
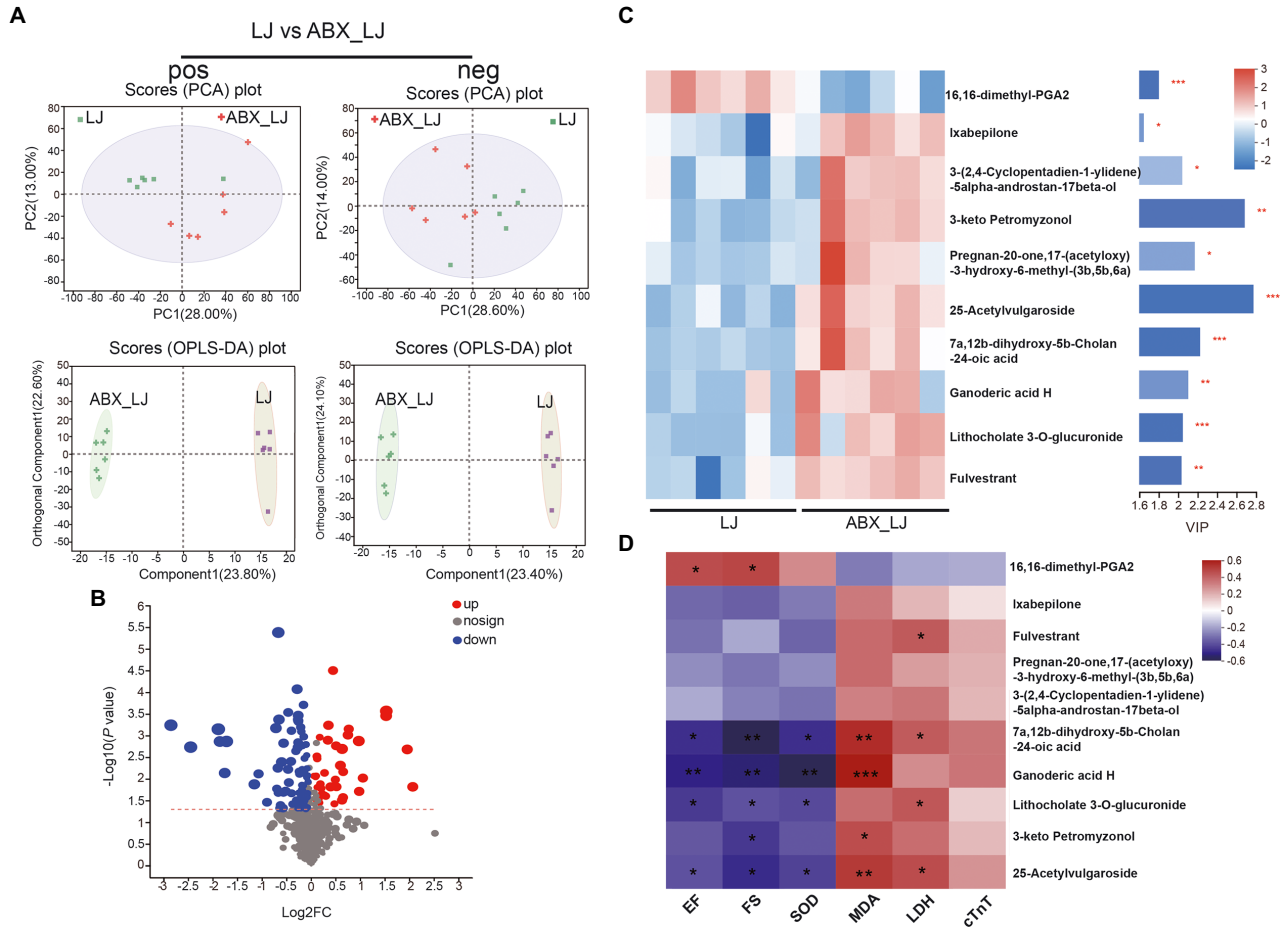
Circulating metabolomics for the quantification of metabolites in AMI rats with ABX and ABX-LJ groups. **(A)** The PCA score plots between LJ and ABX_LJ in positive ion mode [R^2^Xp1 = 0.28, R^2^Xp2 = 0.13, R^2^Xp3 = 0.119] and negative ion mode [R^2^Xp1 = 0.286, R^2^Xp2 = 0.14, R^2^Xp3 = 0.118]. The OPLS-DA score plots between LJ and ABX_LJ in positive ion mode [R^2^X = 0.238, R^2^Y = 0.757, Q^2^ = 0.58] and negative ion mode [R^2^X = 0.234, R^2^Y = 0.731, Q^2^ = 0.553]. **(B)** Volcano plot between LJ and ABX_LJ showing the differentially accumulated [log2 (fold-change) on *x*-axis] and significantly changed [−log10 (*p* value) on y-axis] metabolites. **(C)** The expression profiles of metabolites with VIP > 1 in LJ and ABX_LJ. **(D)** Spearman’s correlation analysis among key efficacy indicators and marker metabolites. ^*^p < 0.05, ^**^p < 0.01, ^***^p < 0.001.

Furthermore, a remarkable separation between the LJ and ABX-LJ samples was also observed in the supervised OPLS-DA score plot, as well as a significant separation of samples between LJ and ABX-LJ groups in the PCA score chart (Figure 6A). Compared with the ABX-LJ treated group, LJ group showed 81 differential metabolites were detected in pos mode, and 41 differential metabolites in neg mode. With VIP > 1 and *p* < 0.05 as the screening condition by ABX, there were top 10 common differential metabolites between LJ and ABX_LJ groups (Figures 6B,C). The level of metabolite 16,16-dimethyl-PGA2 was downregulated, and Ixabepilone, 3-(2,4-Cyclopentadien-1-ylidene)-5alpha-androstan-17beta-ol, 3-keto Petromyzonol, Pregnan-20-one,17-(acetyloxy)-3-hydroxy-6-methyl-(3b,5b,6a), 25-Acetylvulgaroside, 7a,12b-dihydroxy-5b-Cholan-24-oic acid, Ganoderic acid H, Lithocholate 3-O-glucuronide, and Fulvestrant were upregulated after ABX administration (versus LJ) (Supplementary Table S1; Figure 6C). Spearman’s correlation analysis in experiments of *L. johnsonii* administration indicated that 16,16-dimethyl-PGA2 was positively correlated with EF and FS, 7a,12b-dihydroxy-5b-Cholan-24-oic acid, Ganoderic acid H, Lithocholate 3-O-glucuronide, and 25-Acetylvulgaroside were negatively correlated with EF, FS and SOD, while positively correlated with MDA, and 7a,12b-dihydroxy-5b-Cholan-24-oic acid, Lithocholate 3-O-glucuronide, 25-Acetylvulgaroside were positively correlated with LDH (Figure 6D). Further correlation analysis in *L. johnsonii* administration groups revealed that 16,16-dimethyl-PGA2 was positively correlated with *Lactobacillus*, and negatively correlated with *Romboutsia*. Lithocholate 3-O-glucuronide was negatively correlated with *Lactobacillus*, and *norank_f_Muribaculaceae* (Supplementary Figure S4).

## 4. Discussion

The remodeling of the gut microbiota to the beneficial side can delay the pathological exacerbation of CVDs (Chen et al., 2020). Given that the gut microbiota can be modified with a variety of interventions, it can be targeted for the modulation of the host signaling pathways involved in AMI pathogenesis. Gut commensals as functional probiotics for alleviating CVDs are typically claimed to be capable of restoring the gut microbiota and maintaining balance (Hsu et al., 2021; Jin et al., 2021). Some gut-related microbes acting as functional probiotics have been identified by studies on the function in the improvement of cardiac function post-AMI. *L. rhamnosus* administration may have a beneficial effect on cardiac remodeling in patients with AMI (Moludi et al., 2021). *Bifidobacterium* spp. mitigate the pathological effects of AMI in animals. The development of probiotic-based enterobacterial modulation for the alleviation of CVDs is of great importance (Zhao et al., 2021b). And rational combinations of probiotics should provide an alternative to drug treatment in patients in primary cardiovascular disease prevention with mildly added cardiovascular risk and in some statin-intolerant patients (Cicero and Colletti, 2016). Together, the majority of the positive results provided by probiotic treatments should be combined with clinical drug treatment, that is more instructive. This study reveals that the pathological process of AMI and gut microbiota community were improved by the newly isolated *L. johnsonii* over time, suggesting that *L. johnsonii* has a cardioprotective effect and thus clinic value.

The reported biomarkers of the gut dysbiosis of AMI, including high F/B ratio (Wu et al., 2017), pro-AMI microbes, and lower beneficial microbes (Zununi Vahed et al., 2018), can be attenuated by the health benefits of probiotics. We found that the *L. johnsonii* derived through microbial remodeling was characterized by upregulated bacteria, included *Muribaculaceae* and *Lactobacillus*, and the downregulated bacteria include the *Romboutsia*, and *Clostridia* UCG-014. The positive alteration of symbiosis bacteria can reduce the infarcted size, ischemia injury, and inflammation, and they can regulate lipid metabolism and overall cardiac survival. The restoration of gut microbiota community richness and diversity and the beneficial bacterium *Muribaculaceae* contribute to the repression of intestinal barrier dysfunction, inflammation, and disorder of lipid metabolism, and these effects are negatively correlated with AMI development (Wang L. et al., 2021; Zhang et al., 2021). However, some proinflammatory bacteria [e.g., *Clostridia* UCG-014 (Brandsma et al., 2019; Wang Y. et al., 2021)] increased in abundance, inflammation, and upregulation of lipid proinflammatory metabolites occurred, eventually exacerbating AMI. The increased abundance of *Romboutsia* (Zhang et al., 2020; Yang et al., 2021) is associated with high cardiovascular risk.

The gut-heart axis is a novel concept that provides insights into the complex mechanisms of AMI (Zununi Vahed et al., 2018). The intestinal barrier is the key link in the communication routes between gut microbiota and heart (Lewis and Taylor, 2020). The “leakiness” of the intestinal barrier is characterized by the impairment of linker proteins, including occludin and ZO-1, causing the translocation of intestine-derived flora and harmful metabolites (Krack et al., 2005), such as LPS from the cell walls of Gram-negative bacteria, which further triggers systemic inflammation and exacerbates AMI. In this study, we found *L. johnsonii* improved gut barrier integrity, suppressed the production of several oxidative cytokines, pro-inflammatory cytokines, and myocardial injury-related indicators (including SOD, MDA, LDH, cTnT, LPS, and TNF-α), and these effects were accompanied by increased ZO-1 and occludin expression and restoration of cardiac function. Signaling pathways, including Nrf2-Keap1-ARE and AMPK, mediated the effects on the microbiome (Wang et al., 2017; Malik et al., 2018; Lew et al., 2020). Additionally, *Lactobacillus* exerted strong antimicrobial activity against pathogens and reinforced the intestinal barrier, ultimately exerting beneficial effects facilitating gut microbial remodeling and relieving the symptoms of cardiovascular-related diseases (Zhao et al., 2021b). Our results demonstrated common change in cardiac remodeling and colonic pathology after infarction, consistent with the gut-heart axis, a bidirectional relationship between the heart and gut microbiome.

Circulating metabolites derived from gut microbes have undoubtedly helped foster the understanding of therapeutic mechanisms. *L. johnsonii* has beneficial effects that may be attributed to changes in other metabolites in addition to routine metabolites SCFAs and TMAO, such as 16,16-dimethyl-PGA2, and Lithocholate 3-O-glucuronide. *L. johnsonii* treatment diminished the production of cardiac damage metabolites. For example, PGA analogs are potent inhibitors of the anchorage independent growth of murine melanoma cells (Bregman and Meyskens, 1983). Lithocholate 3-O-glucuronide induces cholestasis, causing damage to liver cells and the body (Takikawa et al., 1995). However, more experiments are needed to determine whether or not other metabolites are associated with AMI. The results of the “multi-omics” analysis pointed out various paths forward for the relationships between specific microbial taxa and metabolites for mechanism questions.

Transplantation studies with specific microbial consortia in germ-free or antibiotic-depleted animals facilitate the exploration of the potential role of a specific bacterium in phenotype associations. Antibiotic-treated mice displayed drastic and dose-dependent mortality after AMI, and reduction in antibiotic dosage restored survival in a dose-dependent manner (Garshick et al., 2021). These results were contradictory observations and may be attributed to the biphasic nature of inflammation and the administration of antibiotic doses after AMI (Prabhu and Frangogiannis, 2016). Therefore, we selected the antibiotic dose with high survival and flora clearance for the experiment. Our study showed that antibiotics exerted no severe effects on AMI under the same conditions, suggesting that antibiotics only interfere with the gut microbiota. Antibiotics treatment reduced the *L. johnsonii* abundance, thus allowing the observation of a protective effect of *L. johnsonii* on myocardial infarction. The current treatment of AMI consists mainly of drug administration and surgery, but multiple problems arise during the convalescence of post-AMI (Boersma et al., 2003). Consequently, precision treatments using microbe-targeting interventions have therapeutic potential for AMI.

In conclusion, incorporating multi-omics technologies and standardized *in vivo* strategies alleviated adverse symptoms after AMI by enriching *L. johnsonii* and modulating the gut microbiome to a healthy phenotype. Probiotic development will enhance microbiome intervention and increase understanding of the role of the gut microbiota in diseases.

## Data availability statement

The datasets presented in this study can be found in online repositories. The names of the repository/repositories and accession number(s) can be found at: https://www.ncbi.nlm.nih.gov/, PRJNA732116; https://www.ncbi.nlm.nih.gov/, PRJNA807479.

## Ethics statement

The animal study was reviewed and approved by the Laboratory Animal Ethics Committee of Tianjin University of Traditional Chinese Medicine (TCM-LAEC2019015).

## Author contributions

XqZ performed experiments, analyzed data, and contributed to manuscript drafting. XZ contributed to the data acquisition and analysis, manuscript drafting and revising. YZ and LH contributed to the data analysis. JL and KW contributed to the animal experiments. XG and XW have given final approval of the version to be published and revised the manuscript critically for important intellectual content. All authors read and approved the final manuscript.

## Funding

This study was supported by the National Natural Science Foundation of China (81873104, 81830112, and 81803959); Innovation Team and Talents Cultivation Program of National Administration of Traditional Chinese Medicine (No: ZYYCXTD-C-202009); and Tianjin Municipal Education Commission research project (2022ZD039).

## Conflict of interest

The authors declare that the research was conducted in the absence of any commercial or financial relationships that could be construed as a potential conflict of interest.

## Publisher’s note

All claims expressed in this article are solely those of the authors and do not necessarily represent those of their affiliated organizations, or those of the publisher, the editors and the reviewers. Any product that may be evaluated in this article, or claim that may be made by its manufacturer, is not guaranteed or endorsed by the publisher.

## Footnotes

^1^ http://drive5.com/uparse/

^2^ http://rdp.cme.msu.edu/

^3^ http://www.hmdb.ca/

^4^ https://metlin.scripps.edu/

^5^ http://bioconductor.org/packages/release/bioc/html/ropls.html

## Abbreviations

AB: Alcian blue
AMI: acute myocardial infarction
ATO: atorvastatin
CGMCC: China General Microbiological Culture Collection Center
CK-MB: creatine kinase isoenzymes
CPCC: China Pharmaceutical Culture Collection
cTnT: cardiac troponin-T
EF: ejection fraction
ELISA: Enzyme-linked immunosorbent assay
F/B: Firmicutes to Bacteroidetes
FS: fractional shortening
GC–MS: gas chromatography-tandem mass spectrometry
GM: gut microbiome
H&E: hematoxylin–eosin
HMDB: Human metabolome database
IVS;d: interventricular septal thickness at diastolic
IVS;s: interventricular septal thickness at systole
LAD: left anterior descending coronary artery ligation
LDH: lactate dehydrogenase
LPS: lipopolysaccharide
LV: left ventricular
LVID;d: left ventricular end-diastolic diameter
LVID;s: left ventricular end-systole diameter
MDA: malondialdehyde
OPLS-DA: orthogonal partial least squares discriminate analysis
OTUs: operational taxonomic units
PCA: principle component analysis
PCoA: principal coordinate analysis
SCFAs: short chain fatty acids
SD: Sprague–Dawley
SOD: superoxide dismutase
TMAO: trimethylamine-n-oxide
TNF-α: Tumor necrosis factor -α
UPLC-MS: ultra-performance liquid chromatography–tandem mass spectrometry
VIP: variable importance in the projection
WGA: wheat germ agglutinin
ZO-1: zonula occludens-1
