# Supplementary material for: Remodeling of the gut microbiome by Lactobacillus johnsonii alleviates the development of acute myocardial infarction
Source: Front Microbiol. 2023 Mar 8;14:1140498. doi: 10.3389/fmicb.2023.1140498 (PMC10030800; doi:10.3389/fmicb.2023.1140498)
Supplement: Supplementary file 1 [file Data_Sheet_1.PDF]

Supplementary Material

Remodeling of the gut microbiome by *Lactobacillus johnsonii* alleviates the development of acute myocardial infarction

Xinqin Zhong[1,3], Yucui Zhao[1,3], Lu Huang[1,3], Jiarui Liu[1,3], Kaiyue Wang[1,3], Xiumei Gao[1,3*], Xin Zhao[1,3*] and Xiaoying Wang[1,2,3*]

*** Correspondence:**

Xiumei Gao[1,3*]

gaoxiumei@tjutcm.edu.cn

Xin Zhao[1,3*]

x.zhao26@tjutcm.edu.cn

Xiaoying Wang[1,2,3*]

wxy@tjutcm.edu.cn

1 Supplementary Figures and Tables

Figure S1. (A) EU03 presents antimicrobial activity against *Staphylococcus aureus*, 50 mg/mL apramycin was tested as positive control. (B) EU03 presents G+. (C) The phylogenetic tree constructed by EU03 and *Lactobacillus* species.

Figure S2. Antimicrobial assays of antibiotics mixture against *Lactobacillus* strains. (A) Inhibition zone and (B) bacteriostatic rate against indicator strain *L. johnsonii* EU03; (C) Inhibition zone and (D) bacteriostatic rate against indicator strain *L. rhamnosus* BNCC134266. Bacteriostatic rate (%)=(strain cultivation for 24h OD600nm value - strain cultivation together with antibiotics for 24h OD600nm value)/ strain cultivation for 24h OD600nm value × 100%. The antibiotics mixture including ampicillin (0.25 mg/mL), metronidazole (0.25 mg/mL), neomycin (0.25 mg/mL), and vancomycin (0.125 mg/mL) were dissolved in autoclaved water, and diluted to the concentration of 1/8, 1/16, 1/32, 1/64, 128, 1/256 ratio. The concentration of kanamycin is 50 mg/mL.

Figure S3. Heatmap depicting changes in gut microbial composition at (A) species, and (B) genus levels; (C) Spearman's correlation analysis between 10 identified bacterial species and cardiac traits. *P < 0.05, **P < 0.01, ***P < 0.001.

Figure S4. Interrelationship between gut microbiota composition and host metabolic profile by Spearman correlation analysis in LJ and ABX_LJ. *P < 0.05, **P < 0.01, ***P < 0.001.

Table S1. LJ administration regulates differential metabolites. ("↑" upregulated； "↓" downregulated)

1.1 Supplementary Figures

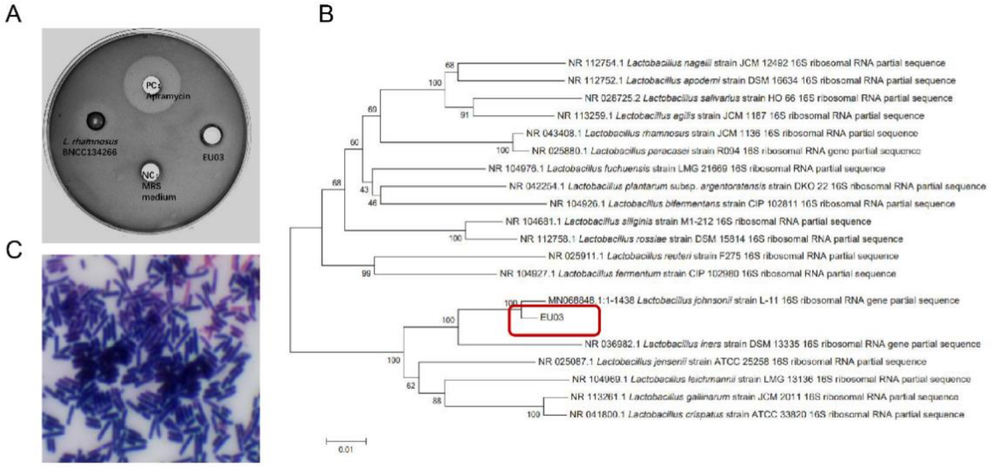

Supplementary Figure 1. (A) EU03 presents antimicrobial activity against *Staphylococcus aureus*, 50 mg/mL apramycin was tested as positive control. (B) EU03 presents G+. (C) The phylogenetic tree constructed by EU03 and *Lactobacillus* species.

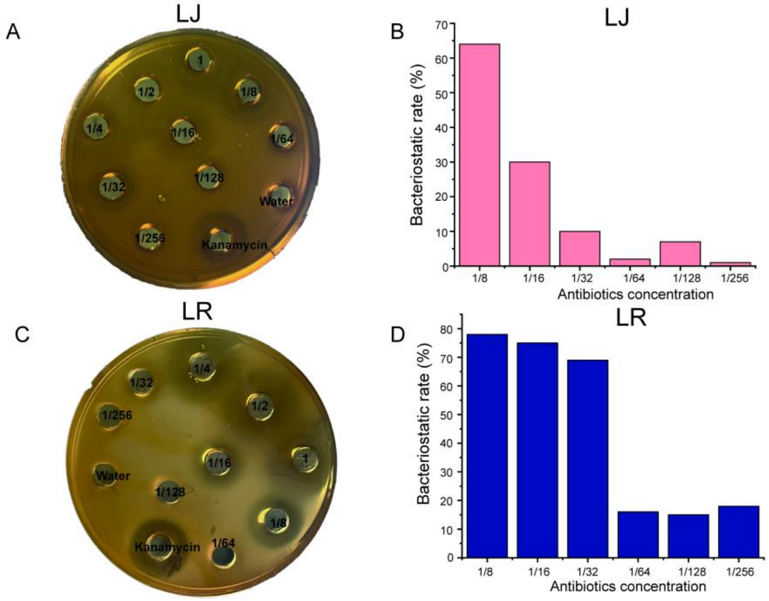

Supplementary Figure 2. Antimicrobial assays of antibiotics mixture against *Lactobacillus* strains. (A) Inhibition zone and (B) bacteriostatic rate against indicator strain *L. johnsonii* EU03; (C) Inhibition zone and (D) bacteriostatic rate against indicator strain *L. rhamnosus* BNCC134266. Bacteriostatic rate (%)=(strain cultivation for 24h OD600nm value - strain cultivation together with antibiotics for 24h OD600nm value)/ strain cultivation for 24h OD600nm value × 100%. The antibiotics mixture

including ampicillin (0.25 mg/mL), metronidazole (0.25 mg/mL), neomycin (0.25 mg/mL), and vancomycin (0.125 mg/mL) were dissolved in autoclaved water, and diluted to the concentration of 1/8, 1/16, 1/32, 1/64, 128, 1/256 ratio. The concentration of kanamycin is 50 mg/mL.

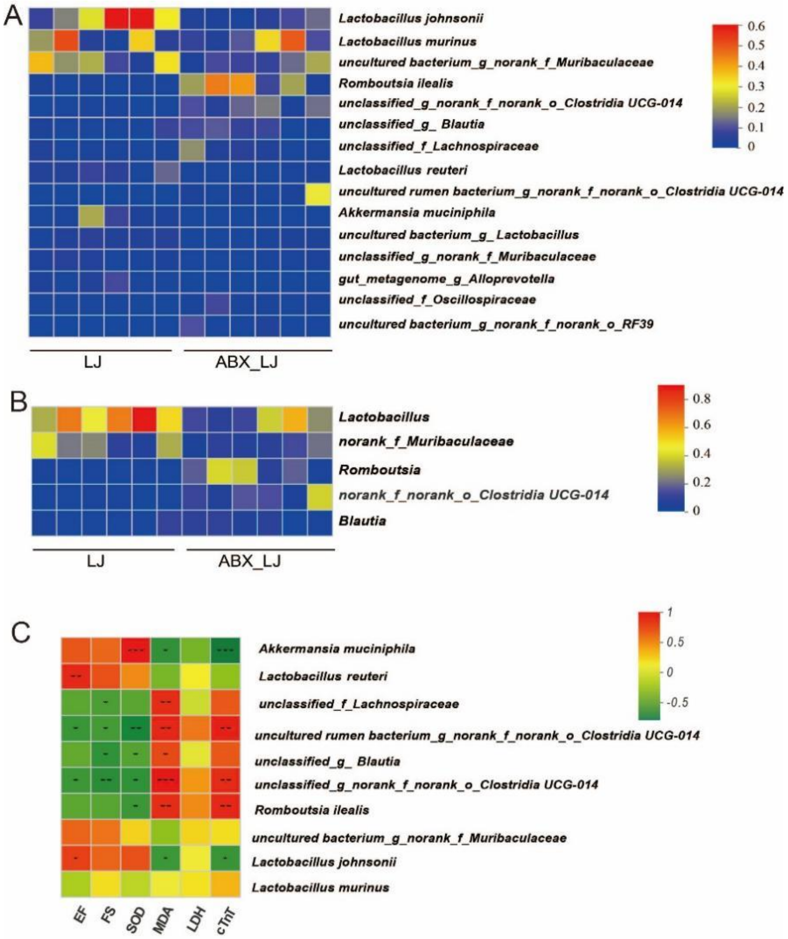

Supplementary Figure 3. Heatmap depicting changes in gut microbial composition at (A) species, and (B) genus levels; (C) Spearman's correlation analysis between 10 identified bacterial species and cardiac traits. **P* < 0.05, ***P* < 0.01, ****P* < 0.001.

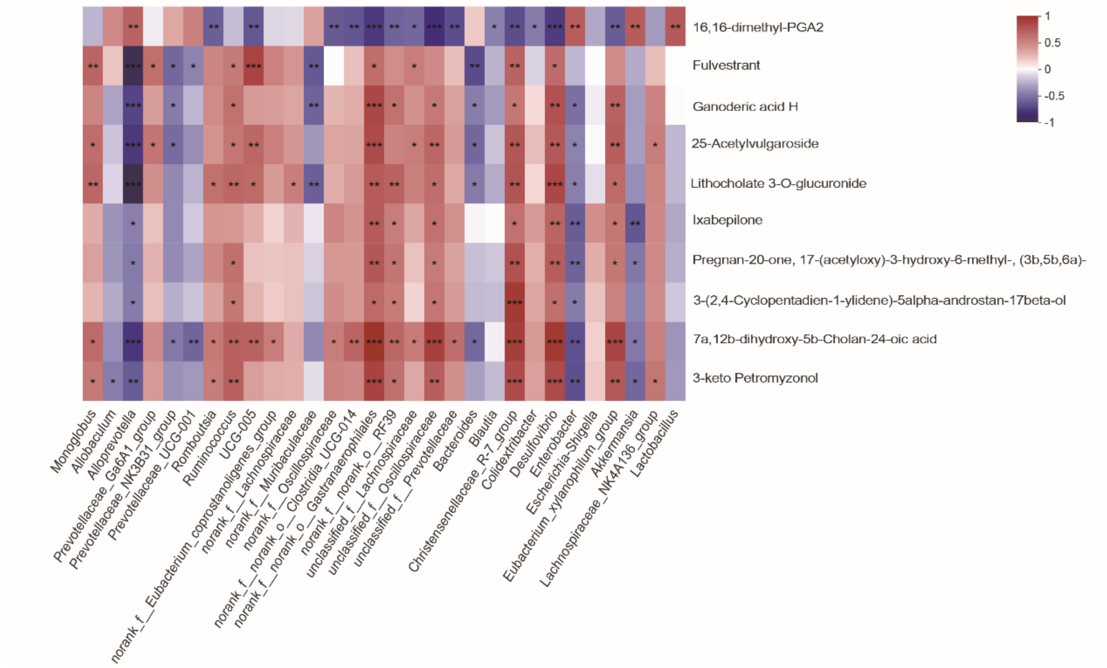

Supplementary Figure 4. Interrelationship between gut microbiota composition and host metabolic profile by Spearman correlation analysis in LJ and ABX_LJ. *$P < 0.05$, **$P < 0.01$, ***$P < 0.001$.

Supplementary Table 1. LJ administration regulates differential metabolites. ("↑"upregulated; "↓"downregulated)

Number	Metabolite	M/Z	Formula	Retention time	
1	Cytidine	266.0743	$C_9H_{13}N_3O_5$	0.6608	↓
2	Creatine	132.0764	$C_4H_9N_3O_2$	0.6327	↓
3	13E-Tetranor-16-carboxy-LTE4	455.1889	$C_{19}H_{27}NO_7S$	0.6705	↓
4	2-O-alpha-D-Galactopyranosyl-1-deoxynojirimycin	367.1502	$C_{12}H_{23}NO_9$	0.7467	↓
5	Phenylacetylglycine	194.0804	$C_{10}H_{11}NO_3$	3.4183	↓
6	3-keto Petromyzonol	375.2888	$C_{24}H_{40}O_4$	6.8045	↓
7	Cinncassiol A 19-glucoside	527.2512	$C_{26}H_{40}O_{12}$	0.5219	↓
8	Pregnan-20-one, 17-(acetyloxy)-3-hydroxy-6-methyl-, (3b,5b,6a)-	391.2842	$C_{24}H_{38}O_4$	6.7745	↓
9	De-O-methylsimmondsin	344.1338	$C_{15}H_{23}NO_9$	1.0143	↓
10	N-(1-Deoxy-1-fructosyl)leucine	276.1437	$C_{12}H_{23}NO_7$	1.1226	↓
11	10-Formyldihydrofolate	472.1575	$C_{20}H_{21}N_7O_7$	2.7574	↑
12	Trans-Grandmarin	310.1302	$C_{15}H_{16}O_6$	3.4278	↑

13	Neoconvallatoxoloside	679.3275	$C_{35}H_{54}O_{15}$	3.5358	↓
14	INDOLE-3-CARBINOL	148.0752	C_9H_9NO	4.131	↓
15	Mono-(3-carboxypropyl) phthalate	291.0272	$C_{12}H_{12}O_6$	4.7061	↑
16	Antibiotic SB 202742	412.2847	$C_{24}H_{34}O_3$	6.2135	↓
17	Piperalol	235.1687	$C_{15}H_{24}O_3$	7.4626	↓
18	PE(20:4(8Z,11Z,14Z,17Z)/ 24:0)	816.6229	$C_{49}H_{90}NO_8P$	11.1039	↓
19	5-Cholestene-3beta,7alpha,12alpha,26-tetrol	435.3468	$C_{27}H_{46}O_4$	8.8839	↑
20	LysoPC(20:4(5Z,8Z,11Z,14Z))	544.3401	$C_{28}H_{50}NO_7P$	8.0762	↓
21	16,16-dimethyl-PGA2	363.2511	$C_{22}H_{34}O_4$	7.3048	↑
22	3-(2,4-Cyclopentadien-1-ylidene)-5alpha-androstan-17beta-ol	339.2676	$C_{24}H_{34}O$	6.8978	↓
23	Prostaglandin E3	315.1950	$C_{20}H_{30}O_5$	6.8443	↑
24	3-Methyl-alpha-ionyl acetate	268.2266	$C_{16}H_{26}O_2$	6.5703	↓
25	Hydroxytorsemide	382.1542	$C_{16}H_{20}N_4O_4S$	5.6062	↓
26	3-Phenylpropyl isovalerate	221.1527	$C_{14}H_{20}O_2$	5.1261	↓
27	Indoxyl	134.0589	C_8H_7NO	3.7066	↓
28	Mabioside C	837.3997	$C_{42}H_{64}O_{14}$	3.5747	↓
29	Fluperamide	577.2469	$C_{30}H_{32}ClF_3N_2O_2$	3.5747	↓
30	Pyridine N-oxide glucuronide	255.0738	$C_{11}H_{14}NO_{7+}$	3.4667	↓
31	1,2,3,4,5,6-Hexahydro-5-(1-hydroxyethylidene)-7H-cyclopenta[b]pyridin-7-one	197.1278	$C_{10}H_{13}NO_2$	3.041	↑
32	Indoleacetaldehyde	160.0752	$C_{10}H_9NO$	2.9839	↑
33	N-(1-Deoxy-1-fructosyl)phenylalanine	328.1388	$C_{15}H_{21}NO_7$	1.9586	↓
34	Niazirinin	339.1548	$C_{16}H_{19}NO_6$	1.5641	↓
35	Serotonin	177.1016	$C_{10}H_{12}N_2O$	1.5079	↓
36	L-Acetylcarnitine	407.2389	$C_9H_{17}NO_4$	0.7178	↓
37	Cytosine	112.0502	$C_4H_5N_3O$	0.6798	↓
38	4-Mercaptobutyl glucosinolate	406.0286	$C_{11}H_{21}NO_9S_3$	7.0603	↑
39	Indoxylsulfuric acid	212.0013	$C_8H_7NO_4S$	3.1826	↓
40	Fulvestrant	587.3021	$C_{32}H_{47}F_5O_3S$	2.9019	↓
41	Ganoderic acid H	609.2503	$C_{32}H_{44}O_9$	2.9689	↓
42	Dihydroferulic acid 4-sulfate	275.0214	$C_{10}H_{12}O_7S$	3.0373	↓
43	4-ethylphenylsulfate	201.0222	$C_8H_{10}O_4S$	4.4365	↑
44	[3-(4-methoxyphenyl)propoxy]sulfonic acid	245.0477	$C_{10}H_{14}O_5S$	4.6428	↑

45	5-(3',4',5'-Trihydroxyphenyl)-gamma-valerolactone-3'-O-sulphate	324.9994	$C_{11}H_{12}O_8S$	4.931	↓
46	25-Acetylvulgaroside	459.2705	$C_{27}H_{42}O_7$	7.1174	↓
47	LysoPE(0:0/20:2(11Z,14Z))	550.3120	$C_{25}H_{48}NO_7P$	7.8028	↑
48	LysoPE(0:0/18:3(9Z,12Z,15Z))	520.2644	$C_{23}H_{42}NO_7P$	8.394	↓
49	PS(18:1(9Z)/0:0)	522.2797	$C_{24}H_{46}NO_9P$	9.1534	↓
50	LysoPE(20:5(5Z,8Z,11Z,14Z,17Z)/0:0)	544.2645	$C_{25}H_{42}NO_7P$	8.5009	↓
51	7a,12b-dihydroxy-5b-Cholan-24-oic acid	437.2882	$C_{24}H_{40}O_4$	7.7935	↓
52	Cepagenin	445.2939	$C_{27}H_{42}O_5$	7.308	↑
53	Ixabepilone	527.2565	$C_{27}H_{42}N_2O_5S$	6.8809	↓
54	Isoaustin	481.1836	$C_{27}H_{32}O_9$	5.7388	↓
55	5'-(3',4'-Dihydroxyphenyl)-gamma-valerolactone sulfate	323.0015	$C_{11}H_{12}O_7S$	4.931	↓
56	2,4-Hexadienyl acetate	121.0664	$C_8H_{12}O_2$	4.4365	↑
57	Indole-3-carboxilic acid-O-sulphate	239.9956	$C_9H_7NO_5S$	2.7478	↓
58	Lithocholate 3-O-glucuronide	587.2993	$C_{30}H_{48}O_9$	0.7507	↓
59	N-(1-Deoxy-1-fructosyl)valine	278.1228	$C_{11}H_{21}NO_7$	0.7507	↓
60	Beta-D-3-Ribofuranosyluric acid	281.0527	$C_{10}H_{12}N_4O_7$	0.7122	↓
